# What research impacts do Australian primary health care researchers expect and achieve?

**DOI:** 10.1186/1478-4505-9-40

**Published:** 2011-11-30

**Authors:** Richard L Reed, Elizabeth C Kalucy, Eleanor Jackson-Bowers, Ellen McIntyre

**Affiliations:** 1 Discipline of General Practice, Flinders University, Bedford Park, Adelaide, Australia; 2 Primary Health Care Research and Information Service, Discipline of General Practice, Flinders University, Bedford Park, Adelaide, Australia

## Abstract

**Background:**

Funding for research is under pressure to be accountable in terms of benefits and translation of research findings into practice and policy. Primary health care research has considerable potential to improve health care in a wide range of settings, but little is known about the extent to which these impacts actually occur. This study examines the impact of individual primary health care research projects on policy and practice from the perspective of Chief Investigators (CIs).

**Methods:**

The project used an online survey adapted from the Buxton and Hanney Payback Framework to collect information about the impacts that CIs expected and achieved from primary health care research projects funded by Australian national competitive grants.

**Results and Discussion:**

Chief Investigators (CIs) provided information about seventeen completed projects. While no CI expected their project to have an impact in every domain of the framework used in the survey, 76% achieved at least half the impacts they expected. Sixteen projects had published and/or presented their work, 10 projects included 11 doctorate awards in their research capacity domain. All CIs expected their research to lead to further research opportunities with 11 achieving this. Ten CIs achieved their expectation of providing information for policy making but only four reported their research had influenced policy making. However 11 CIs achieved their expectation of providing information for organizational decision making and eight reported their research had influenced organizational decision making.

**Conclusion:**

CIs reported that nationally funded primary health care research projects made an impact on knowledge production, staff development and further research, areas within the realm of influence of the research team and within the scope of awareness of the CIs. Some also made an impact on policy and organizational decision-making, and on localized clinical practice and service delivery. CIs reported few broader economic benefits from their research. Routine use of an instrument of this type would facilitate primary health care research funders' determination of the payback for funding of research in this sector.

## Background

How research is developed, conducted, disseminated and assessed is changing. Knowledge translation is high on government agendas and investment in research is expected to yield returns in the form of an improved health system delivering better health outcomes. This expectation raises the level of interest in assessing research impact [1,2] which presents conceptual methodological and practical challenges to the broad and diverse field of primary health care research [3].

Foremost among the research assessment frameworks developed by different groups has been the Payback Framework [4], developed by the Health Economics Research Group from Brunel University which encompasses a diverse range of research outputs and outcomes. Through interviews with CIs of research projects and key informants, detailed narrative case studies of the impact of individual research projects can be developed. This framework has been used in case study [4] and questionnaire format [5] to review the outcomes of funding by the UK Arthritis Research Campaign (ARC), and in questionnaire format, to assess the impact of research funded by the North Thames office of the UK NHS [6] and publicly funded health service research in Hong Kong [7]. The Framework is the basis of assessment of research impact in Canada [8]. Recently the Payback Framework has been the basis of a series of tools used by RAND Europe in an internet survey to map the ARC portfolio and the impacts of this research [5]. It has also been used in health technology assessment research projects in UK (e.g. the National Health Service Health Technology Assessment Programme and the National Coordinating Centre for Health Technology Assessment) in Canada [9] and in the Netherlands [10].

None of the above studies focus specifically on primary health care research, which is notably diverse in its topics, populations, methodologies and settings [11]. Primary health care research is a small but growing field compared with other areas of medical research in Australia [12]. Since 2000 the Australian Government has specifically funded primary health care research capacity building [3], demonstrating its strong interest in primary health care research which is relevant to and informs policy and practice in Australia. This makes it important to find a way to assess the impact of primary health care research.

An initial study on the impact of Australian primary health care research was used to understand the feasibility of using the Payback Framework. Case studies of four primary health care research projects in Australia were compiled from documentary evidence and telephone interviews with CIs and key informants, leading to the conclusion that use of the Framework and associated logic model made it feasible to determine the impacts of primary health care research with some modifications [3].

This paper reports on the second stage of the study, which aimed to assess the impact of a larger sample of primary health care research projects and to explore how impact was achieved. This paper presents an overview of the expected and achieved impacts of these primary health care research projects. An analysis of pathways by which this impact was achieved will be published separately. The Flinders University Social and Behavioural Ethics Committee approved the project.

## Methods

### Questionnaire design

The Buxton and Hanney Payback Framework [4] consists of a logical model of the research process and definition of payback criteria. Building on the experience of the first stage of this study [3], we adapted the Buxton and Hanney Payback Framework [4] by adding a new domain of impact which assessed enhanced capacity for research transfer. This includes both improved university engagement with the community and the health care sector and enhanced pathways and relationships for research transfer to policy makers, organisational decision makers, practitioners and consumers. This domain was added as this has been a major area of emphasis by the Australian Government and has been the primary basis for explicitly funding research in the primary care sector. An extra topic was added on the specific impact of research on clinical practice. Additionally the types of outputs were broadened to include presentations, websites, and media as they are being increasingly accepted as valid alternative outputs particularly for clinical and policy impact (as opposed to traditional research impact).

Based on the understanding that the research team's mission is relevant to how and to what extent the impact should be evaluated [13], the final questionnaire included a dichotomous screening question to determine if impact in each category was expected by the research team, and a subsequent dichotomous question to indicate whether it was achieved.

As knowledge production was treated as an essential output rather than an impact, CIs were surveyed only about the number of publications or presentations achieved from the research, likewise the number of doctorates achieved through staff development.

The final draft of the questionnaire was piloted with a project officer whose project was included in trial but who did not participate in the completion of the questionnaire. This questionnaire was ultimately adapted to an on-line format. A list of questions used in the online survey is listed in Appendix 1.

### The sample

The sample included all primary health care research projects funded competitively by the National Health and Medical Research Council (NHMRC), the Aboriginal Health Medical Research Council, the General Practice Evaluation Program (GPEP), the Cooperative Research Centre for Aboriginal Health (CRCAH) and the Primary Health Care Research Evaluation and Development Strategy (PHCRED) to a minimum of $80, 000, commencing from 2000 and due for completion by 2006. The possible sample consisted of 59 primary health care research projects, but accurate contact details were only available for CIs of 41 projects despite an extensive search. Data collection was initiated in October 2007 and the survey was open for 6 weeks.

CIs were contacted by email and provided with a link to an information sheet about the project and a link to an on-line questionnaire. A consent form was incorporated into the questionnaire. A follow up email was sent two weeks later and, where a telephone number was available; non-responders were also contacted by telephone if they did not respond to the email requests.

### Analysis

Potential categories of impact were designated in five domains: research transfer; research targeting, capacity building and absorption; informing policy and product development; health and health sector benefits; and broader economic benefits. In each category a variable was computed indicating whether an impact was expected but not achieved, expected and achieved, or achieved but not expected. An impact measure of the percentage of expected impacts achieved was computed for each project.

## Results

**Figure 1:**
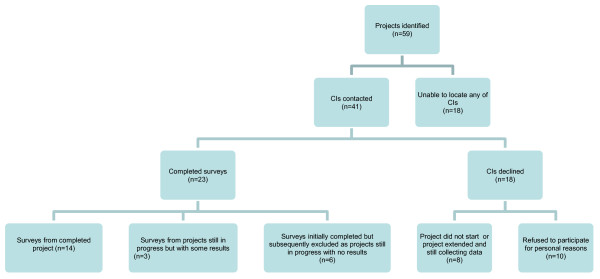
**Participation flow chart**.

Of the 41 projects where the CI was contactable, 23 surveys were returned of which a further 6 were excluded from analysis as the project was still in progress at the time of contact with no results to date. The results of 17 projects are reported (see Figure 1). There were 12 intervention projects and 5 descriptive projects. Specific aspects of the research projects of the 17 respondents are listed in Table 1.

**Table 1:** Research Projects Included in Study

Project Name	Last Year of Funding	Funding Source	Amount In AUD	Status
A rapid literature summary service to enhance evidence-based clinical decision in general practice*	2000	GPEP	$109, 000	C

Program of resource, information and support for mothers: a community randomised trial*	2001	NHMRC	$549, 000	C

Shared care for serious mental illness: caring for carers	2003	GPEP	$93, 000	C

Randomised controlled trial of physiotherapy injections, saline injections and exercises in the treatment of chronic low back pain*	2003	GPEP	$99, 000	C

The evidence-based consumer: making informed decisions about menopause, hormone replacement and complementary therapies*	2004	PHCRED	$97, 000	C

Cognitive screening in General Practice*	2005	NHMRC	$300, 000	C

Threats to patient safety in general practice: Investigating errors in Australian primary healthcare*	2005	NHMRC	$80, 000	I

A randomised controlled trial of physiotherapy and corticosteroid injections of lateral epicondylagia in primary care*	2005	NHMRC	$190, 000	C

Audit and Best Practice in Chronic Disease*	2005	AHMRC CRCAH	$747, 403	C

A randomised controlled trial of a decision aid for prenatal screening and diagnosis*	2005	NHMRC	$269, 000	C

Screening for chlamydia trachomatis with routine Pap smears in general practice: A randomised controlled trial*	2005	NHMRC	$350, 000	C

Systematic practice-based asthma care in the Australian Setting*	2006	NHMRC	$563, 000	C

Disclosure and attitudes to lesbians: Outcomes in General Practice (DIALOG)	2006	NHMRC	$426, 000	I

Doctors, their patients and computers: The new medical consultation - a study of the impact of computerisation	2006	NHMRC	$103, 000	I

Learning from Action*	2006	CRCAH	$244, 214	C

Impact of socioeconomic disadvantage on chronic disease management in primary care: A diabetes case study	2006	NHMRC	$258, 000	C

Urban locational disadvantage and health: compositional and contextual determinants	2006	NHMRC PHCRED	$608, 000	C

AHMRC = Aboriginal Health and Medical Research Council

CRCAH = Cooperative Research Council for Aboriginal Health

GPEP = General Practice Evaluation Program

NHMRC = National Health and Medical Research Council

* Intervention Study, no star indicates descriptive study

C = Complete at time of survey

I = Incomplete but results available at time of survey

CIs reported producing 39 peer-reviewed publications from their projects. Two projects yielded seven publications each, while four had not yet published in journals at the time of the survey in October 2007. The projects that produced four or more publications were either large scale complex studies involving multiple partners, or clinical trials.

The large range of journals in which primary health care research is published is shown by the 39 peer reviewed publications being published in 26 separate journals. Journal Impact Factors were available for the journals in which 22 of the 39 articles were published. With the exception of two articles, most articles were published in journals with Impact Factors less than 3.0.

Researchers reported many outputs for their research in addition to peer reviewed publications although not as many as they intended. Projects resulted in conference presentations (15/17 projects), presentations to potential users (14/16), media stories (12/16) newsletter articles (12/16), reports (11/17) and project websites (10/17). Most of these outputs involved considerable effort on the part of the research team.

### Impacts: expected and achieved

CIs reported on impacts they expected and which they perceived their project had achieved, in each domain and category. The broad array of impacts achieved was almost entirely in the categories that CIs expected, but the number of impacts was less than they expected. Additional File 1, Table S1 shows the expected and achieved impacts, the impact score which is the proportion of expected impacts achieved in each domain, category and project as well as the number of papers, conference presentations and doctorates from each project.

Overall, 76% (13/17 projects) of CIs considered they had achieved at least half the impacts they expected. The impact score ranged from 93% (13/14 expectations were achieved in Project 1) to 17% (2/12 expectations were achieved in Project 17). No CI expected their project to have an impact in every domain of the impact framework used in the survey. Three projects had an unexpected impact.

CIs considered they achieved all or nearly all expectations in enhanced relationships for research transfer and was used in guideline development or systematic review (100%, 6/6 projects and 3/3 projects respectively), and staff development (94%, 15/16 projects). Further examination of the data showed that in two of the three cases the systematic review was written by the research team themselves as a side study to their projects.

All seventeen CIs expected their research to lead to further research opportunities, the only category in which they had common expectations, and 65% reported achieving this.

Three quarters of CIs expected their research to inform policy development, organizational decision making and education. The impact score was considerably higher for providing information for policy making (77%) than for influencing policy making (31%), However, CIs reported high impact scores both in providing information for (85%) and influencing organizational decision making (73%).

In terms of health and health sector benefits, CIs reported most impact in improved service delivery (70%), use in clinical practice (58%) and improved health outcomes (50%). Examination of the survey data suggests that these perceived impacts affected the health service organizations, clinicians and patients who took part in the research projects.

Impact scores were low or zero in a few categories - more equitable service delivery (25%), cost savings (0%), intellectual property gains (0%), improvements in population health (10%), and other economic (10%) or social (0%) impacts.

## Discussion

The use of an online questionnaire in this research impact study provided data on a wide range of perceived impacts of seventeen nationally funded primary health care research projects. By asking CIs to identify areas in which they expected their projects to make an impact, and then identify areas where they had achieved impact, the study has shown that all but four of the seventeen projects in the sample reported achieving more than half of the impacts they expected. The sample is not large enough to draw any conclusions about the relative proportion of impacts achieved by intervention or descriptive types of research projects.

The CIs of these projects report numerous and diverse perceived impacts, and nearly all of the potential impacts noted in the framework occurred at least once. The projects performed strongly by achieving impacts on further research and staff development, and research transfer.

Over 90% of projects generated further research opportunities and staff development, both of which are critical for the ongoing growth and development of the primary health care research sector in Australia. Developing capacity for research transfer by enhancing university engagement with user groups and enhancing relationships for research transfer is another important area of impact, indicating that researchers are aware of the importance of these processes to complement their strong dissemination efforts through papers, conferences and other media.

The proportion of projects achieving expected impacts was over 70% for providing information for policy and organizational decision making, influencing organizational decision-making, and over 50% for use in clinical practice and improved service delivery. This reflects a high level of engagement of the researchers with potential users of their research findings.

The substantial impact on education (69%, 9/16 projects achieved this) underscores the strong link between research and education at Australian Universities.

Research use depends not only on the quality of the research and the transmission process, but on the dynamics of the political context and there is no guarantee that high quality research will be used [1]. It is therefore not surprising that most impact is achieved in areas, which are under the researchers' control, such as in knowledge production, further research, or in the organizations involved with the project.

The fact that 31% of CIs achieved their expectation of influencing policy development is notable. Providing information for policy and for organizational decision-making, an area where CIs achieved high proportions of expected impacts (77% and 85%), is to some extent dependent on the skills and exertion of the research team, but influencing policy is usually dependent on many factors, including the dynamics of the political context [14-17]. The reported impacts on policy from these seventeen projects were substantial. Some examples were the study of the 3+ Asthma Plan in the Systematic practice-based asthma care in the Australian Setting project, which contributed to a new model of Medicare Benefits Scheme item numbers for asthma, while the Audit and Best Practice in Chronic Disease project informed the development of major Indigenous Health initiatives (Continuous Improvement projects and the Healthy for Life Program). It also influenced Northern Territory policy making as all primary care services are now expected to adopt the approaches developed in the project. The approach has also been adopted in Western Australia, New South Wales, Queensland and South Australia. The Disclosure and Attitudes to Lesbians: Outcomes in General Practice (DIALOG) project findings contributed to a Victorian Government action plan and cultural awareness training for general practitioners (Table 1).

The results in terms of influence on organizational decision-making, and flow on effects on localised clinical practice and service delivery are consistent with an earlier Australian study of NHMRC funded projects commissioned by the National Institute of Clinical Studies (NICS) which found 24% had resulted in some kind of translation into practice, usually in the researcher's local area or health service [14]. Little impact was expected or reported on broader economic benefits, and none on cost savings or intellectual property gains.

### Limitations

Limitations are the small number of projects, the Chief Investigators' knowledge of their project impacts, and risks of a bias towards positive benefits [5].

We did not expect that we would be unable to locate CIs of 18 of the 59 projects, despite extensive searching for these individuals. Due to limitations in data available from the Australian Government on funded grants, a detailed analysis of patterns of non-response could not be undertaken. The sample frame consisted of research directly funded by the Australian Government provided by competitive peer review with minimum funding of $80, 000. These results cannot be generalised to primary health care research funded through other means such as tenders, non-competitive processes, consultancies, for lesser amounts and by other organizations including service providing organizations, and professional associations [18].

In an earlier study we collected qualitative data through interviews with CIs and gathered copious information that provided more context to better understand the results. However this process was time-consuming for both the interviewee and interviewer and so unlikely to be a useful tool for routine use by funders. The use of an online questionnaire in this subsequent study took less time but yielded information which was less detailed.

Some modification of the survey may increase the accuracy of reporting. The use of dichotomous variables to assess categories of impact could more accurately be replaced by a scale depicting a range of levels of achievement (e.g. significant, some, marginal impact).

The results are likely to underestimate the impacts of these projects, as CIs do not usually know of the use made of their work beyond publication in the literature and events in their own realm of awareness. As three projects were yet to be fully completed at the time of the survey, and others were very recently completed and were still writing for publication, it is possible that further impact would be achieved over time. The collective impact of the projects is thus a work in progress and must be viewed as a snapshot and not a definitive statement of eventual impact.

### Implications for primary health care research

This study demonstrates that individual primary health care research projects can have an impact in a range of areas addition to traditional production of knowledge through journal articles and reports.

An ongoing process to identify the impacts of individual research projects could raise awareness and understanding of some research impacts and how they are achieved. Researchers who could identify the impacts of their work could then use this knowledge to illustrate the relevance of their work as part of their ongoing engagement with the people in their particular sector on whose good will they often depend. However, the current Excellence in Research Australia initiative provides more incentives for academic researchers to publish in highly rated journals than to identify or achieve societal impact [19].

Proving a connection between individual research projects and impacts such as more equitable service delivery, cost savings or improvements in population health is unlikely, because of the difficulties of attribution, the need for synthesis of findings of many primary studies and the complex social and political processes through which policy change takes place. A focus on individual studies may result in underestimating the impact of primary health care research.

## Conclusions

This study shows that CIs perceived their individual primary health care research projects had made numerous impacts on research transfer, knowledge production, research capacity building, informing policy and localised health and health sector benefits, the categories of the Payback framework. Such impacts are consistent with the interest of the Australian government in funding relevant primary health care research that can inform policy and practice. The impacts were in the areas expected by the Chief Investigators, though fewer than they expected.

The use of an online questionnaire was practical for collecting information retrospectively from CIs about the impact of their research, while recognising that the information they provide is limited to the impacts within their realm of awareness, and time since completion. Routine use of such a questionnaire by primary health care funders at the completion of a funded project and two years after completion of the project would allow funders to better determine value for money. This is particularly relevant as specified funding is generally justified by the critical importance of research to have an impact on the primary health care sector which in comparison to the hospital sector has had a small fraction of funding through traditional sources. It would in turn encourage investigators to keep more accurate records regarding the impacts of their research on policy and practice at the time that they become aware of these impacts and to broaden their focus beyond publications and citations.

## Competing interests

Authors received funding from the PHCRED Strategy, as did two of the projects in the study.

## Authors' contributions

RLR: assisted with the analysis and interpretation of the data and contributed to the manuscript; ECK: assisted with analysis and interpretation of the data and contributed to the manuscript; EJB: conducted the survey and analysis of the data and contributed to the manuscript; EM: assisted with analysis and interpretation of the data and contributed to the manuscript; All authors have read and approved the final manuscript.

## Appendix 1: Questions Asked in the Online Survey

### Project Detail

1 Project title

2 Please provide a brief description of your research project, its methodology, main findings and how these have been applied.

3 Research Opportunities

3.1 Did you expect your research findings to lead to other research opportunities?

3.2 Did your research findings lead to other research opportunities?

3.3 Please describe what occurred and comment on how this came about.

4 Professional Development

4.1 Did you expect staff development, educational benefits or higher degrees to be earned as a result of your project?

4.2 Were there staff development, educational benefits or higher degrees earned as a result of your project?

4.3 Please describe what occurred and comment on how this came about.

5 State or Australian Government Policy Making

5.1 Did you intend your research findings to provide information relevant to State or Australian Government policy making?

5.2 Did your research findings provide information relevant to State or Australian Government policy making?

5.3 Please describe what occurred and comment on how this came about.

5.4 Did you intend your research findings to influence State or Australian Government policy making?

5.5 Did your the research findings influence State or Australian Government policy making?

5.6 Please describe what occurred and comment on how this came about.

6 Organisational, Local, Regional Level Decision Making

6.1 Did you intend your research findings to provide information relevant to organisational, local or regional level decision making?

6.2 Did your research findings provide information relevant to organisational, local or regional level decision making?

6.3 Please describe what occurred and comment on how this came about.

6.4 Did you intend your research to influence organisational, local or regional level decision making?

6.5 Did your research findings influence organisational, local or regional level decision making?

6.6 Please describe what occurred and comment on how this came about.

7 Education Curricula or Training Policies

7.1 Did you expect your research findings to influence education curricula or training policies?

7.2 Did your research findings influence education curricula or training policies?

7.3 Please describe what occurred and comment on how this came about.

7.4 Did you expect your research findings to be included in practice guidelines or in a systematic review?

7.5 Were your research findings included in practice guidelines or in a systematic review?

7.6 Please tell us which one (s).

8 Clinical Practice

8.1 Did you expect your research findings to be used in clinical practice?

8.2 Were your research findings used in clinical practice?

8.3 Please describe what occurred and comment on how this came about.

9 Service Delivery

9.1 Did you expect your research findings to lead to improvements in the process of service delivery?

9.2 Did your research findings lead to improvements in process of service delivery?

9.3 Please describe what occurred and comment on how this came about.

10 Service Improvement

10.1 Did you expect your research findings to lead to more equitable allocation of resources, better targeting of services or improved access to services?

10.2 Did your research findings lead to more equitable allocation of resources, better targeting of services or improved access to services?

10.3 Please describe what occurred and comment on how this came about.

11 Cost Reduction

11.1 Did you expect your research findings to lead to cost reduction in the delivery of existing services?

11.2 Did your research findings lead to cost reduction in the delivery of existing services?

11.3 Please describe what occurred and comment on how this came about.

12 Health Outcomes

12.1 Did you expect your research findings to lead to improved health outcomes for individuals or groups?

12.2 Did your research findings lead to improved health outcomes for individuals or groups?

12.3 Please describe what occurred and comment on how this came about.

13 Better Health at a Population Level

13.1 Did you expect your research findings to contribute to better health at a population level?

13.2 Did your research findings contribute to better health at a population level?

13.3 Please describe what occurred and comment on how this came about.

14 Intellectual Property Rights

14.1 Did you expect there to be any revenues gained from intellectual property rights?

14.2 Were there any revenues gained from intellectual property rights?

14.3 Please describe what occurred and comment on how this came about.

15 Product Development

15.1 Did you expect your research findings to inform product development?

15.2 Did your research findings inform product development?

15.3 Please describe what occurred and comment on how this came about.

16 Economic Benefits

16.1 Did you expect any other economic benefits?

16.2 Were there any other economic benefits?

16.3 Please describe what occurred and comment on how this came about.

17 Other Social, Environmental, Economic or Cultural Benefits

17.1 Did you expect your research findings to lead to any other social, environmental, economic or cultural benefits?

17.2 Were there any other social, environmental, economic or cultural benefits?

17.3 Please describe what occurred and comment on how this came about.

18 Research Transfer

18.1 Did you expect your project to lead to enhanced relationships for research transfer to potential users (eg. policy makers, organisational decision makers, practitioners and consumers)?

18.2 Did your project lead to enhanced relationships for research transfer to potential users (eg. policy makers, organisational decision makers, practitioners and consumers)?

18.3 Please describe what occurred and comment on how this came about.

19 University Engagement

19.1 Did you expect your research findings or processes to lead to improved university engagement with the community and the health care sector?

19.2 Did your research findings or processes lead to improved university engagement with the community and the health care sector?

19.3 Please describe what occurred and comment on how this came about.

20 Unfavourable Circumstances

20.1 Were there any circumstances which were not favourable for the use of your research findings?

20.2 Please describe what occurred and comment on how this came about.

21 Engagement with Potential Users

Did you involve potential users of your research in

21.1 clarifying project aims?

21.2 designing or refining methods?

21.3 interpretation of findings?

21.4 disseminating findings?

21.5 How successful was the involvement of your potential users in achieving use of your research findings?

22 Pathways to Use

22.1 How important were your professional networks in achieving use of your research findings?

22.2 A person of influence (possibly yourself) may be instrumental in bringing your research findings to a decision making forum. How important was the involvement of a person of influence in achieving use of your research findings?

22.3 How important were chance encounters or serendipitous events in achieving use of your research findings?

22.4 Were there any other activities, events, organisations or processes that facilitated the use of your research findings?

22.5 Please describe what occurred and comment on how this came about.

23 Peer Reviewed Publications

23.1 How many peer reviewed publications resulted from your project?

23.2 Please list peer reviewed publications from your project in this box.

23.3 To your knowledge have the peer reviewed publications been influential in achieving use of your research findings?

24 Conference Presentations

24.1 How many conference presentations were made by team members in order to raise awareness of your project, your methods or to disseminate your research findings?

24.2 Please list conference presentations in this box.

24.3 To your knowledge were these conference presentations influential in achieving use of your research findings?

25 Other Presentations

25.1 How many other presentations were made by team members to policy makers, practitioners and decision makers to raise awareness of your project, your methods or to disseminate your research findings?

25.2 To your knowledge were these presentations influential in achieving use of your research findings?

26 Media Releases

26.1 How many media releases were made about the project?

26.2 To your knowledge were these media releases influential in achieving use of your research findings?

27 Newsletter Articles

27.1 How many newsletter articles resulted from your project?

27.2 To your knowledge were these newsletter articles influential in achieving use of your research findings?

28 Other Media

28.1 How many media stories, appearances or interviews were there about your project?

28.2 To your knowledge were these media events influential in achieving use of your research findings?

29 Final Report

29.1 Was there a final report publicly available?

29.2 To your knowledge was your report influential in achieving use of your research findings?

30 Project Website

30.1 Was your project featured on a website/webpage?

30.2 To your knowledge was this website or webpage influential in achieving use of your research findings?

31 Publications or resources

31.1 Were there other publications or resources produced?

31.2 Please describe what was produced.

31.3 To your knowledge were these publications or resources influential in achieving use of your research findings?

32 Other Modes of Dissemination

32.1 Were there any other modes of dissemination that were important in achieving use of your research

findings?

32.2 Please describe the other modes of dissemination.

32.3 To your knowledge were there other modes of dissemination influential in achieving use of your research findings?

## Supplementary Material

Additional file 1 **Table S1 - Chief Investigators' perceptions of expected and achieved impact of Australian primary health care research projects**. Click here for file
