# Supplementary material for: What research impacts do Australian primary health care researchers expect and achieve?
Source: Health Res Policy Syst. 2011 Nov 30;9:40. doi: 10.1186/1478-4505-9-40 (PMC3256100; doi:10.1186/1478-4505-9-40)
Supplement: Additional file 1 — Table S1 - Chief Investigators' perceptions of expected and achieved impact of Australian primary health care research projects. [file 1478-4505-9-40-S1.DOCX]

| **Table S1: Chief Investigators’ perceptions of expected and achieved impact of Australian primary health care research projects.** | | | | | | | | | | | | | | | | | | | | | | | | | | |
| --- | --- | --- | --- | --- | --- | --- | --- | --- | --- | --- | --- | --- | --- | --- | --- | --- | --- | --- | --- | --- | --- | --- | --- | --- | --- | --- |
| Project | Research Transfer | | Knowledge Production | | Research capacity building | | | Informing policy and product development | | | | | | | Health and health sector benefits | | | | | | Broader economic benefits | | | Totals by project | | |
|  | Enhanced university engagement with user groups | Enhanced relationships for research transfer | Number of peer reviewed publications | Number conference presentations | Further research  opportunities | Number PhDs | Staff development | Provided information for  policy making | Influenced policy making | Provided information for organizational decision making | Influenced organizational decision making | Was used in education | Informed product development | Was used in guideline development or systematic review | Used in clinical practice | Led to improvements  in service delivery | Led to more equitable  service delivery | Led to cost savings | Led to improved health outcomes | Intellectual property gains | Led to improvements in population health | Other economic impacts | Other social impacts | Number impacts expected | Number impacts achieved | % expectations fulfilled |
| Project 1* | ● |  | 4 | 16 | ● | 1 | ● | ● | ● | ● | ● | ● |  |  | ● | ● | ● |  | ● |  | ● | ○ |  | 14 | 13 | 93 |
| Project 2 | ● |  | 1 | 5 | ○ | 1 | ● | ! | ● | ● | ● | ● |  |  | ● | ○ |  |  | ● |  | ○ |  | ○ | 12 | 9 | 75 |
| Project 3* | ● |  | 4 | 7 | ○ |  | ● | ● | ○ | ● | ● | ● |  | ● |  | ! | ○ |  | ○ |  | ○ |  | ● | 13 | 9 | 69 |
| Project 4* | ● | ● | 1 | 1 | ● |  | ● | ● | ○ | ● | ○ | ● |  |  | ● | ● | ● |  | ● |  | ○ | ○ | ○ | 16 | 11 | 69 |
| Project 5* |  |  | 5 | 30 | ● | 1 | ● | ○ | ○ | ● | ● | ○ |  | ● | ● |  |  |  |  |  |  |  |  | 9 | 6 | 67 |
| Project 6* | ○ |  | 1 | 2 | ○ |  | ● | ● | ● | ● | ● | ● |  |  | ○ |  |  |  |  |  |  |  |  | 9 | 6 | 67 |
| Project 7 |  |  | 2 | 5 | ○ | 1 | ● | ● | ○ | ● | ! | ○ |  |  |  |  |  |  |  |  |  |  |  | 6 | 4 | 67 |
| Project 8 | ● | ● | 0 | 5 | ● |  | ● | ● | ○ | ● | ● | ● | ● |  |  | ○ | ○ |  |  |  | ○ |  | ○ | 14 | 9 | 64 |
| Project 9* |  |  | 0 | 0 | ● |  | ● | ○ | ○ | ● | ● |  |  |  | ● | ● | ○ |  | ● |  |  |  | ○ | 11 | 7 | 64 |
| Project 10 | ● | ● | 7 | 13 | ● | 2 | ● | ● | ○ | ● | ○ |  |  |  |  | ● | ○ |  |  |  | ○ |  |  | 11 | 7 | 64 |
| Project 11* | ○ |  | 7 | 16 | ● | 1 | ● |  |  |  |  | ● |  | ● | ● |  |  |  | ○ |  |  | ○ |  | 8 | 5 | 63 |
| Project 12* | ○ |  | 2 | 9 | ○ | 1 | ● | ○ | ○ |  |  | ● |  |  | ● | ● |  |  | ● | ○ |  |  |  | 10 | 5 | 50 |
| Project 13* | ● | ● | 1 | 7 | ● |  | ● | ● | ● |  |  | ● |  |  | ○ | ○ | ○ |  | ○ |  | ○ | ○ | ○ | 14 | 7 | 50 |
| Project 14* |  | ● | 3 | 6 | ● | 1 | ○ | ● | ○ | ● | ○ |  | ○ |  | ○ | ● |  |  |  |  | ○ |  | ○ | 12 | 5 | 42 |
| Project 15* |  |  | 0 | 0 | ● |  |  |  |  |  |  |  | ● |  | ○ |  |  |  |  | ○ |  | ○ | ○ | 6 | 2 | 33 |
| Project 16* | ○ | ● | 1 | 4 | ○ | 1 | ● |  |  | ○ | ○ | ○ | ○ |  |  |  |  |  | ○ |  | ○ |  |  | 10 | 2 | 20 |
| Project 17 | ○ |  | 0 | 5 | ● | 1 | ● | ○ |  | ○ |  | ○ |  |  | ○ | ○ | ○ |  | ○ |  | ○ |  | ○ | 12 | 2 | 17 |
| No. Impacts expected | 12 | 6 |  |  | 17 |  | 16 | 13 | 13 | 13 | 11 | 13 | 4 | 3 | 12 | 10 | 8 | 0 | 10 | 2 | 10 | 5 | 9 |  | | |
| No. Impacts achieved | 7 | 6 | 39 | 131 | 11 |  | 15 | 10 | 4 | 11 | 8 | 9 | 2 | 3 | 7 | 7 | 2 | 0 | 5 | 0 | 1 | 0 | 1 |  |  |  |
| Legend: ○ Project was expected to have impact in this category ● Project was expected to have impact and perceived achieved impact in this category  * Intervention studies; the remainder were descriptive studies. ! Unexpected impact | | | | | | | | | | | | | | | | | | | | | | | | | | |
